# Atomistic insights into the degradation of perfluorosulfonic acid membranes: A reactive force field molecular dynamics study

**DOI:** 10.1371/journal.pone.0346636

**Published:** 2026-04-07

**Authors:** Wanhua Qi, Yang Li, Shifeng Ruan, Xian Gui, Yaoli Xu, Haibin Lu

**Affiliations:** 1 College of Computer Science and Information Engineering, Anyang Institute of Technology, Anyang, China; 2 Flight College, Anyang Institute of Technology, Anyang, China; 3 Software Engineering College, Zhengzhou University of Light Industry, Zhengzhou, China; Donghua University, CHINA

## Abstract

The chemical degradation of perfluorosulfonic acid (PFSA) membranes remains a critical barrier to the commercial viability of proton exchange membrane fuel cells (PEMFCs). This study employs reactive force field molecular dynamics (ReaxFF-MD) simulations to elucidate the atomistic-scale mechanisms of degradation initiated by hydroxyl (·OH) and hydrogen (H·) radicals. It is demonstrated that H· radicals preferentially abstract fluorine atoms from the polymer backbone (–CF₂–) and attack terminal carboxyl groups (–COOH), thereby initiating unzipping reactions that result in chain scission and defluorination. In contrast, ·OH radicals predominantly attack sulfonic acid groups (–SO₃H) and tertiary carbon sites, leading to side-chain cleavage with minimal backbone disruption. Mixed radical environments exhibit synergistic degradation kinetics, wherein ·OH-mediated regeneration of H· radicals substantially accelerate membrane failure. Elevated temperatures further exacerbate degradation by reducing activation barriers and enhancing radical mobility. A fundamental trade-off between dielectric properties and chemical stability is identified: although polar functional groups enhance proton conductivity, they simultaneously introduce sites susceptible to radical degradation. Selective passivation of –COOH groups is shown to significantly enhance durability while retaining high proton conductivity. These insights provide a mechanistic foundation for the rational design of degradation-resistant PFSA membranes.

## 1. Introduction

Proton exchange membrane fuel cells (PEMFCs) represent one of the most promising clean energy conversion technologies, offering high energy efficiency, zero direct emissions, and robust performance across a wide range of operating temperatures. At the heart of PEMFC operation lies the perfluorosulfonic acid (PFSA) membrane, which serves the dual function of conducting protons from anode to cathode while preventing the crossover of reactant gases (hydrogen and oxygen) [1,2]. The most widely used PFSA membrane, Nafion®, consists of a hydrophobic polytetrafluoroethylene (PTFE) backbone with pendant perfluorinated vinyl ether side chains terminated by sulfonic acid groups (-SO₃H), which provide the proton conductivity essential for fuel cell operation [3,4].

Despite the exceptional chemical stability of perfluorinated polymers, PFSA membranes undergo progressive degradation during fuel cell operation, ultimately limiting device lifetime and hindering commercial deployment [5,6]. This degradation is primarily chemical in nature, initiated by reactive oxygen species (ROS) generated during electrochemical reactions. During normal operation, incomplete oxygen reduction at the cathode and hydrogen oxidation at the anode can generate hydrogen peroxide (H₂O₂), which subsequently diffuses into the membrane [7]. In the presence of trace metal ion contaminants (Fe²⁺, Cu²⁺), H₂O₂ decomposes via Fenton-type reactions to produce highly reactive radicals including hydroxyl radicals (·OH), hydroperoxyl radicals (·OOH), and hydrogen radicals (H·) [8,9].

These radical species attack the PFSA polymer structure through distinct pathways. Four primary degradation mechanisms have been identified in the literature: (i) ·OH radical attack on carboxylic acid end-groups initiating the well-known “unzipping” mechanism; (ii) ·OH attack on carbon-sulfur (C-S) bonds in the sulfonic acid groups; (iii) ·OH attack on ether (C-O-C) linkages in the side chains; and (iv) H· attack on tertiary carbon-fluorine (C-F) bonds in the polymer backbone [2]. The unzipping mechanism, first reported by Curtin et al. and subsequently refined by Coms, involves sequential abstraction of hydrogen from carboxylic end-groups by ·OH radicals, leading to chain scission and the release of hydrogen fluoride (HF) and carbon dioxide (CO₂) [10,11]. This process regenerates carboxylic acid groups, allowing the degradation cycle to continue until the polymer chain is completely consumed.

Importantly, recent studies have highlighted the previously underappreciated role of H· radicals in membrane degradation [12,13]. Within the hydrogen-rich anode environment of the fuel cell, ·OH radicals can be converted to H· radicals through the reaction: H₂ + ·OH → H· + H₂O [14,15]. Unlike ·OH radicals, which primarily abstract hydrogen atoms, H· radicals can abstract fluorine atoms from tertiary C-F bonds on the polymer backbone, forming thermodynamically stable HF due to the greater bond dissociation strength of H-F compared to C-F [12]. Liquid chromatography/mass spectrometry (LC/MS) analysis of fuel cell effluent water has provided compelling experimental evidence for this pathway, revealing partially hydrogenated side chain fragments such as CH₃-CF₂CF₂CF₂-SO₃H that can only arise from H·-mediated reduction processes [13]. Furthermore, density functional theory (DFT) calculations have shown that the activation barrier for H· attack on tertiary carbon (~84 kJ/mol) is significantly lower than that for ·OH attack on ether linkages (~250 kJ/mol), suggesting that H· radicals may play a more prominent role in degradation than previously recognized [3,12].

Despite significant advances in understanding PFSA degradation mechanisms, several critical knowledge gaps remain. Current models do not adequately quantify the relative contributions of H· versus ·OH attack pathways, nor do they capture the potential synergistic or competitive interactions when multiple radical species are present simultaneously. Moreover, while experimental techniques such as LC/MS can detect degradation products, they cannot provide the mechanistic resolution required to distinguish between competing reaction pathways at the molecular level [13]. Atomistic simulations offer a powerful complementary approach to address these limitations.

In this study, we employ reactive force field molecular dynamics (ReaxFF-MD) simulations to develop a comprehensive understanding of radical-induced PFSA membrane degradation. ReaxFF-MD enables the modeling of complex bond-breaking and bond-forming processes in large-scale systems under realistic reactive conditions [14–16]. Specifically, our simulations aim to: (i) quantify the reaction mechanisms, kinetics, and relative contributions of H· and ·OH attacks on critical PFSA structural components including the backbone, sulfonic acid groups, and carboxylic acid end-groups; (ii) elucidate the atomistic pathways and energetic determinants of H·-initiated tertiary fluorine abstraction leading to backbone scission; and (iii) investigate synergistic and competitive effects arising from the concurrent presence of H· and ·OH radicals. By providing fundamental atomistic insights into the predominant degradation mechanisms, this work aims to inform the rational design of more durable PFSA membrane materials for next-generation fuel cells.

## 2. Simulation details

### 2.1 Overview of ReaxFF-MD

The ReaxFF-MD method is a bond-order-based reactive force field specifically designed to simulate chemical reactions in complex molecular systems [17]. It is grounded in the concept of continuously updated bond orders, derived from instantaneous interatomic distances. Unlike conventional fixed- topology force fields that require predefined bond connectivity, ReaxFF dynamically calculates bond orders during simulations, enabling accurate description of bond formation and dissociation processes [17–20].

The total energy in ReaxFF is partitioned into multiple contributions, each dependent on bond orders and atomic charges (Eq. 1):


$$E_{system}=E_{bond}+E_{over}+E_{under}+E_{val}+E_{pen}+E_{tors}+E_{conj}+E_{vdW}+E_{Coulomb}$$ (1)

where $E_{bond}$ represents the covalent bond energy, $E_{over}$ and $E_{under}$ are over- and under-coordination correction terms, $E_{val}$ is the valence angle energy, $E_{pen}$ is a penalty term for two double bonds sharing an atom, $E_{tors}$ is the torsion angle energy, $E_{conj}$ accounts for conjugation effects, and $E_{vdW}$ and $E_{Coulomb}$ describe non-bonded van der Waals and Coulombic interactions, respectively [14,17].

The dynamic assignment of bond orders and charges allows ReaxFF-MD to naturally simulate complex reaction pathways—including the radical reactions critical to degradation studies. Combined with its computational efficiency relative to quantum mechanical methods, ReaxFF-MD serves as a powerful tool for exploring chemical transformations in large molecular systems such as polymers under reactive conditions. Simulations are commonly conducted in the canonical (NVT) or isothermal-isobaric (NPT) ensembles, using thermostats (e.g., Berendsen, Nosé-Hoover) and barostats for temperature and pressure control [21,22].

### 2.2 Model construction

All ReaxFF-MD simulations were performed using the Amsterdam Modeling Suite (AMS) software package (version 2022.102). The reactive force field employed in this work was constructed by building upon a series of previously validated ReaxFF parameterizations, which has been validated for fluorocarbon and sulfonic acid chemistry relevant to PFSA membrane systems [23–25].

**Fig 1:**
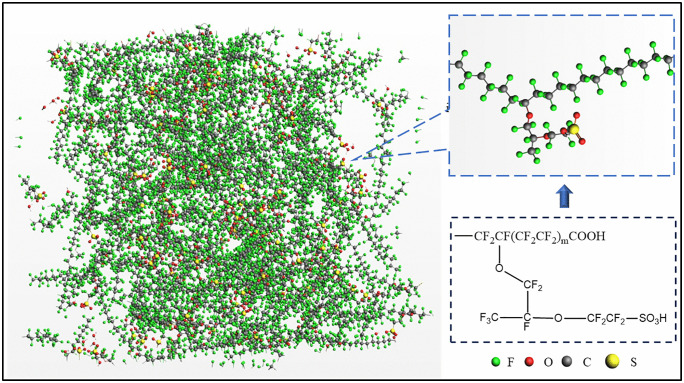
Representative snapshot of the PFSA model comprising 100 polymer chains in the initial configuration.

The PFSA model structure features a polytetrafluoroethylene (PTFE) backbone with perfluorinated vinyl ether side chains, terminated with carboxyl (–COOH) groups to represent end-group chemistry characteristic of partially stabilized membranes (Fig 1). To reduce computational cost, polymer chains with only 20 repeating units were constructed; the functional groups present in these model chains are comparable to those of commercial Nafion® membranes. The initial simulation cell was constructed from 100 such polymer chains (Fig 1).

The simulation protocol proceeded as follows. Periodic boundary conditions were applied in all three dimensions to simulate bulk membrane behavior. After initial geometry optimization using the conjugate gradient algorithm (energy convergence: 1.0 × 10⁻ ⁵ kcal/mol), the system was equilibrated using a Berendsen thermostat (100 fs damping) and barostat (500 fs damping) at 1 atm. A subsequent 100 ps relaxation at 300 K with a 0.25 fs time step yielded stable initial configurations for reactive simulations. The equilibrated density of 0.720–0.727 g/cm³ is lower than that of dry Nafion (~1.98 g/cm³) due to the high hydration level (λ ≈ 15–20) and thermal expansion at elevated simulation temperatures, but is consistent with values typically observed for highly hydrated PFSA membranes.

To systematically examine the chemical behavior of radical species, four distinct simulation models were constructed by embedding specific radicals into the polymer network using the Packmol algorithm [26]: (i) Model A: containing hydroxyl radicals (·OH) only; (ii) Model B: containing hydrogen radicals (H·) only; (iii) Model C: a mixed system containing both ·OH and H· radicals; (iv) Model D: a radical-free control system. The radical concentrations in Models A-C were set at 100 radicals per simulation cell, corresponding to a concentration of approximately 0.1 mol/L, which represents accelerated degradation conditions compared to typical fuel cell operation. All models underwent full energy minimization followed by NPT equilibration to ensure structural integrity.

A multiscale comparative analysis was additionally performed using both polymer-embedded systems (Table 1) and corresponding vacuum-isolated systems containing identical radical species, in order to distinguish intrinsic radical reactivity from polymer environmental effects. Each system was equilibrated under NVT ensemble conditions using a Berendsen thermostat (temperature damping constant = 100 fs) and an integration time step of 0.25 fs. A 50 ps equilibration run was first performed at each target temperature to ensure sufficient system relaxation and attainment of thermodynamic equilibrium. Subsequently, 250 ps production runs were performed at 300 K, as well as at five additional temperatures ranging from 283 K to 363 K. (with the exception of Model D, for which a 20 ps production run was conducted), followed by data collection. It should be noted that the elevated simulation temperatures are necessary to observe statistically significant degradation events within the simulation timescale; the Arrhenius analysis presented in Section 2.5 enables extrapolation to experimentally relevant temperatures.

**Table 1:** Details of the MD models constructed for the PFSA degradation study.

Model	Num. of PFSA chains	Num. of •OH	Num. of •H	Density (g/cm³)	Cell dimension (Å)
Model A	100	100	0	0.727	74.99
Model B	100	0	100	0.721	74.99
Model C	100	50	50	0.724	74.99
Model D	100	0	0	0.720	74.99

### 2.3 Polarizability and dielectric constant calculation

The molecular polarizability was determined using the AMS software suite, which employs quantum mechanical methods to evaluate electronic response properties. Calculations were performed on isolated PFSA oligomer structures extracted from equilibrated ReaxFF configurations. The high-frequency dielectric constant was then derived from the Clausius–Mossotti equation, which relates the dielectric constant (ɛ) to the molecular polarizability (𝛼) as follows (Eq. 2):


$$\frac{\varepsilon -1}{\varepsilon +2}=\frac{N_{A}\rho \alpha }{3M}$$ (2)

where $N_{A}$ is Avogadro's number, *ρ* is the polymer density, *M* is the molecular weight of the repeating unit, and *α* is the calculated molecular polarizability.

### 2.4 DFT energy calculations

Density functional theory (DFT) calculations were conducted using the Amsterdam Density Functional (ADF) module in the AMS software suite to evaluate the energetics of key elementary reactions. The PBE functional with a triple-zeta polarized (TZP) basis set was employed, and scalar relativistic effects were treated via the ZORA method. Reaction energies were computed for representative bond cleavage events identified in Section 3.2, including ·OH-mediated side-chain detachment, H·-initiated C-F bond scission, and H· regeneration reactions. These calculations provided thermodynamic validation for the proposed degradation mechanisms.

### 2.5 Reaction rate analysis

The degradation kinetics were analyzed by tracking the decrease in the number of intact PFSA chains and specific functional groups (including C–S bonds, C–O–C ether linkages, and –COOH end-groups) over time. Bond cleavage events were identified using a bond-order cutoff of 0.3, below which atoms were considered non-bonded. Under conditions of excess radical concentration, the degradation follows pseudo-first-order kinetics, and the reaction rate constant (k) can be determined using Eq. 3:


$$ln\frac{N_{\text{0}}}{N_{t}}\;=kt$$ (3)

where *N*_*0*_ and *N*_*t*_ represent the number of intact functional groups at time zero and time *t*, respectively.

According to the Arrhenius equation, the temperature dependence of the rate constant is described by Eq. 4:


$$k=Aexp(-\frac{E_{a}}{RT}\;)$$ (4)

where *A* is the pre-exponential factor, $E_{a}$ is the activation energy, R is the gas constant (8.314 J·mol⁻¹·K⁻¹), and T is the absolute temperature.

Taking the natural logarithm of both sides yields the linearized form Eq. 5:


$$\text{ln}(k)=\text{ln}(A)-\frac{E_{a}}{RT}$$ (5)

Thus, the activation energy can be extracted from the slope of the ln(*k*) versus 1/T plot. Simulations were performed at five different temperatures for this purpose [10].

## 3. Results and discussion

### 3.1 Radical-specific degradation kinetics and bond cleavage analysis

#### 3.1.1 Evolution of radical populations and mechanistic implications.

**Fig 2:**
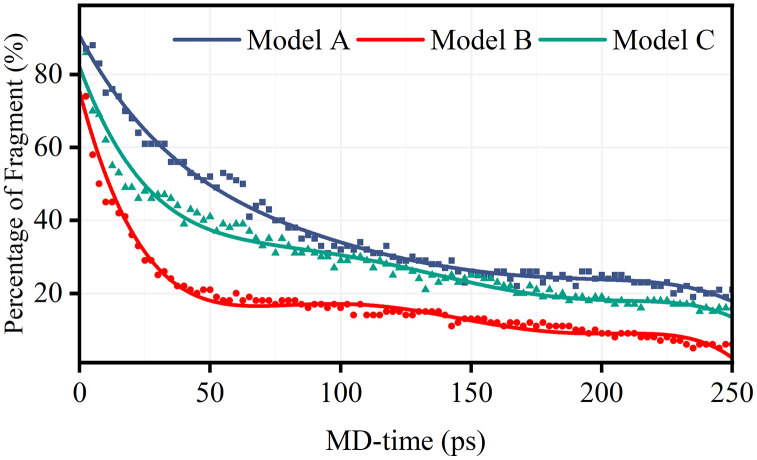
Temporal evolution of unreacted polymer segments in the Model A (•OH-only), Model B (H•-only), and Model C (mixed •OH/H•) systems at 300 K.

**Figure 2** illustrates the temporal evolution of unreacted segments across the three radical environments (Models A–C), providing critical insights into degradation kinetics and radical interactions within PFSA membranes. Understanding these temporal profiles is essential for elucidating the fundamental degradation mechanisms that limit membrane durability in practical fuel cell applications [27].

In the Model A system, the high retention of unreacted segments (>95% at 250 ps) is consistent with the selective attack of ·OH radicals on sulfonic acid groups (–SO₃H) and tertiary carbons, where regenerative pathways such as radical transfer through peroxide intermediates limit net consumption. This observation is consistent with previous DFT studies showing that ·OH radicals preferentially target electron-rich sites in PFSA structures [2].

In contrast, the Model B system exhibits rapid depletion of unreacted segments (~80% consumed within 250 ps), consistent with the high reactivity of H· toward backbone –CF₂– moieties and –COOH terminal groups. This behavior corroborates H·-initiated unzipping as a key degradation route. The thermodynamic driving force for H· attack on C–F bonds originate from the significantly greater bond dissociation energy of H–F (568 kJ/mol) compared to that of C–F bonds (approximately 485 kJ/mol), making fluorine abstraction energetically favorable [12,28]. This mechanism was first proposed by Ghassemzadeh et al. and has subsequently been confirmed through LC/MS analysis of fuel cell effluent water [13].

#### 3.1.2 Radical consumption kinetics and degradation pathways.

**Fig 3:**
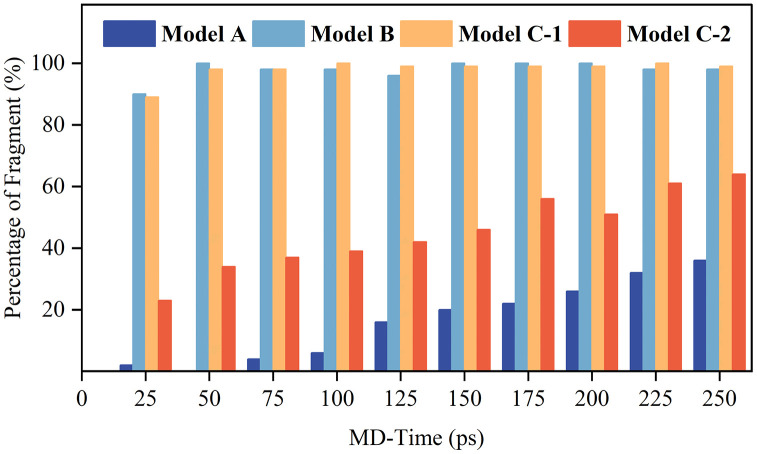
Temporal profiles of radical concentrations in the Model A (·OH-only), Model B (H·-only), and Model C (mixed ·OH/H·) systems. Within Model C, Model C −1 and Model C −2 denote the ·OH and H· populations, respectively at 300 K.

Figure 3 illustrates the temporal evolution of radical concentrations in the Model A, Model B, and Model C systems, providing mechanistic insights into the degradation profiles observed in **Fig 2**. For clarity of notation: Model C-1 denotes the ·OH radical population within Model C, and Model C-2 denotes the H· radical population within Model C. This distinction is important because the behavior of each radical species in the mixed system differs significantly from that in the corresponding isolated single-radical system.

The nearly invariant ·OH population in Model A (>95% retention at 250 ps) confirms its catalytic role in side-chain cleavage: ·OH radicals preferentially oxidize sulfonic acid groups or abstract hydrogen atoms from tertiary carbons, generating stabilized intermediates that regenerate ·OH via peroxide decomposition pathways, thereby minimizing net radical consumption. This catalytic behavior is consistent with the mechanism proposed by Zatoń et al. [29].

In contrast, Model B exhibits rapid H· decay (~80% consumption within 250 ps), resulting from irreversible fluorine abstraction from backbone –CF₂– units and hydrogen abstraction from carboxylic terminal groups. Each H· reaction generates carbon-centered polymer radicals that initiate β-scission or defluorination—chain reactions consuming multiple H· radicals per fragmentation event. The unzipping mechanism proceeds through sequential carbon elimination from the polymer chain ends (see Reaction Scheme 1 in Section 3.2) [2].

The Model C system displays biphasic kinetics. During the initial phase (0–100 ps), rapid H· consumption resembles that of Model B, followed by a stabilization plateau (100–175 ps) indicative of compensatory H· regeneration through the interconversion pathway H₂ + ·OH → H· + H₂O, which has been identified as a key mechanism for maintaining H· concentrations in the hydrogen-rich anode environment of operating fuel cells [30]. The sustained H· concentration during the plateau phase prolongs backbone attack compared to Model B, while the delayed ·OH decay reflects initial competitive suppression by H·. Early-stage H· attack produces carbon-centered radicals that react with permeated H₂ to regenerate H·, while ·OH radicals oxidize newly formed –CFH– sites to peroxyl radicals that can decompose and regenerate ·OH via Russell termination [31,32]. Such synergistic radical cycles enhance irreversible chain scission and establish self-propagating degradation pathways, highlighting the critical role of radical interconversion in accelerating membrane failure. Similar synergistic effects have been reported in accelerated stress testing studies, where combined chemical and mechanical stresses produce degradation rates exceeding the sum of individual contributions [33,34].

#### 3.1.3 Ether linkage fragmentation as the backbone degradation signature.

**Fig 4:**
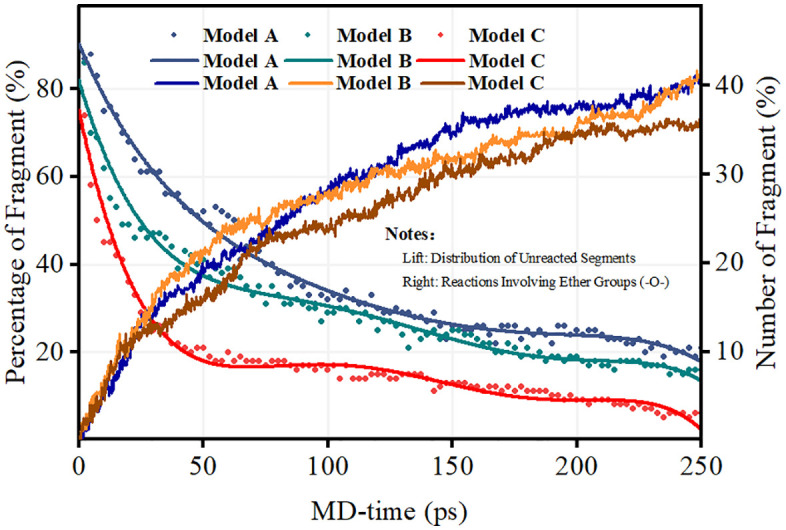
Radical-specific ether linkage cleavage profiles in the Model A, Model B, and Model C systems at 300 K.

The cumulative cleavage of ether groups (-O-), as quantified in Fig 4, offers direct evidence of radical-specific scission mechanisms within the PFSA backbone. The ether linkages (C–O–C) connecting the side chains to the main backbone represent critical structural elements whose integrity determines overall membrane mechanical properties [35].

The Model B system exhibits exponential growth in ether bond cleavage (>12 bonds by 250 ps), underscoring the unique capacity of H· radicals to initiate reactions at α-carbon sites adjacent to ether oxygen atoms. The resulting carbon-centered radical undergoes rapid β-scission, leading to irreversible backbone fragmentation (see Reaction Scheme 2 in Section 3.2). The β-scission mechanism has been confirmed through DFT calculations showing low activation barriers (approximately 40–60 kJ/mol) for this process [36].

In contrast, Model A demonstrates negligible ether bond cleavage (<3 bonds), consistent with the preferential targeting of sulfonic acid groups and tertiary carbon sites by ·OH radicals rather than ether-linked carbons. The selectivity of ·OH radicals for sulfonic acid groups has been attributed to the high electron density at sulfur centers, which attracts electrophilic ·OH radicals [12,25].

Notably, the Model C system exhibits biphasic cleavage kinetics. During Phase I (0–100 ps), relatively slow ether scission (~4 bonds) is observed, reflecting suppressed H·-initiated events due to competition with ·OH for reactive sites. In Phase II (100–250 ps), cleavage accelerates markedly, coinciding with ·OH-mediated regeneration of H· radicals—particularly at void interfaces (Fig 7)-where localized high H· concentrations drive further attack on residual ether linkages. This localized concentration effect has been observed in experimental studies using spatially resolved fluoride mapping techniques [30].

**Fig 5:**
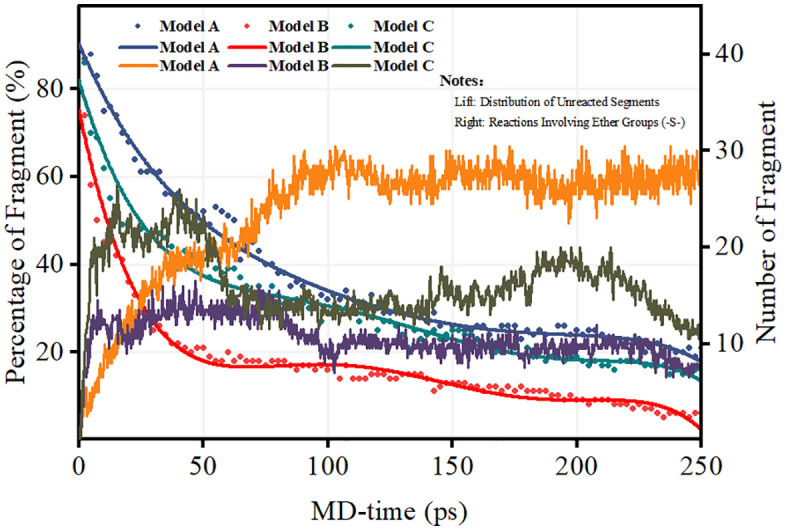
Temporal evolution of sulfur-containing bond cleavage during radical-mediated degradation in the Model A, Model B, and Model C systems at 300 K.

**Fig 6:**
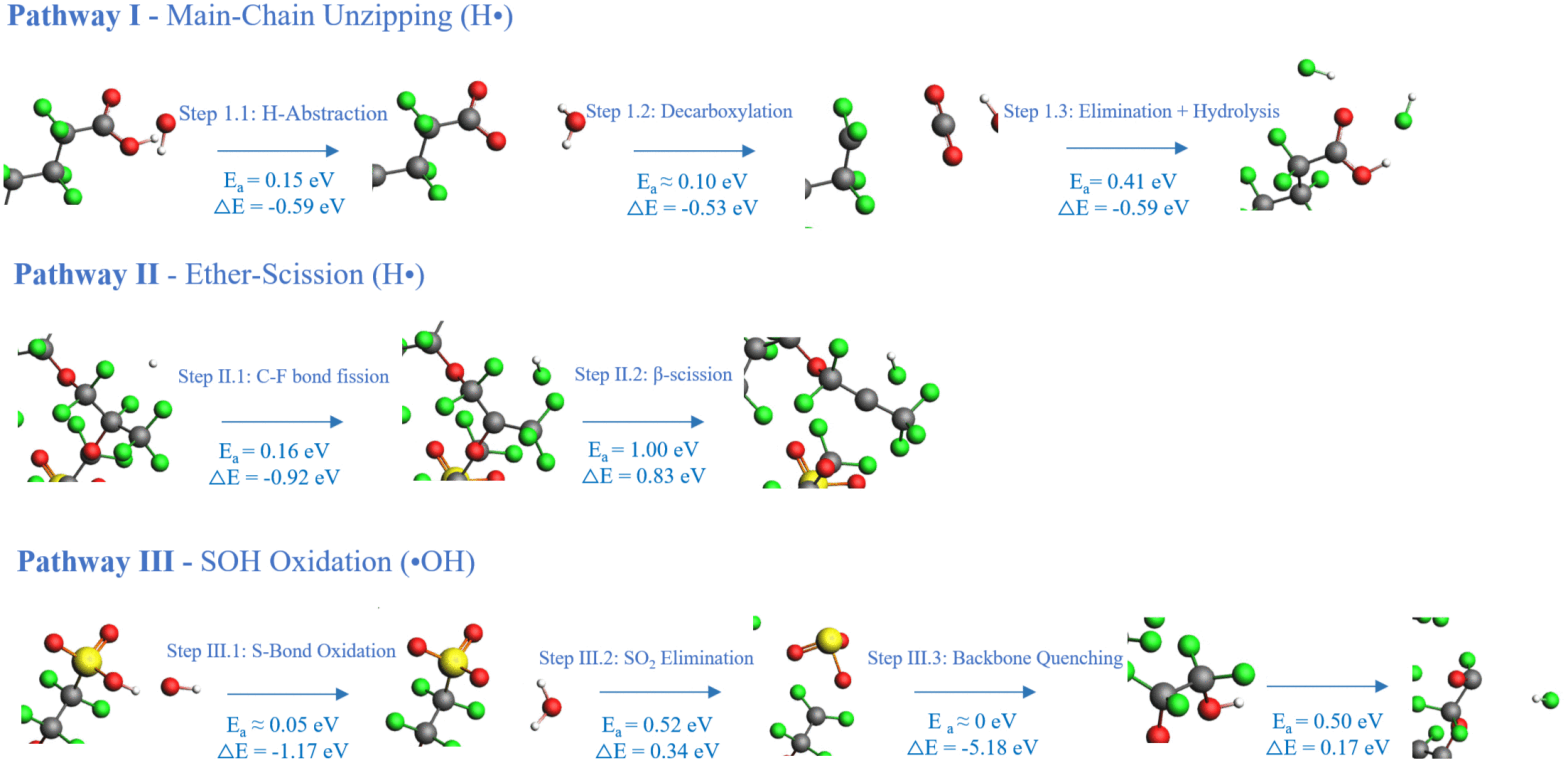
Unified radical-mediated PFSA degradation pathways.

#### 3.1.4 Sulfur-containing group degradation: competing pathways and radical selectivity.

As illustrated in Fig 5, the fragmentation profiles of sulfur-containing groups reveal radical-specific degradation mechanisms that complement the ether bond cleavage patterns described in Section 3.1.3. The sulfonic acid groups (–SO₃H) are essential for proton conductivity, and their loss directly impacts fuel cell performance [33].

**Fig 7:**
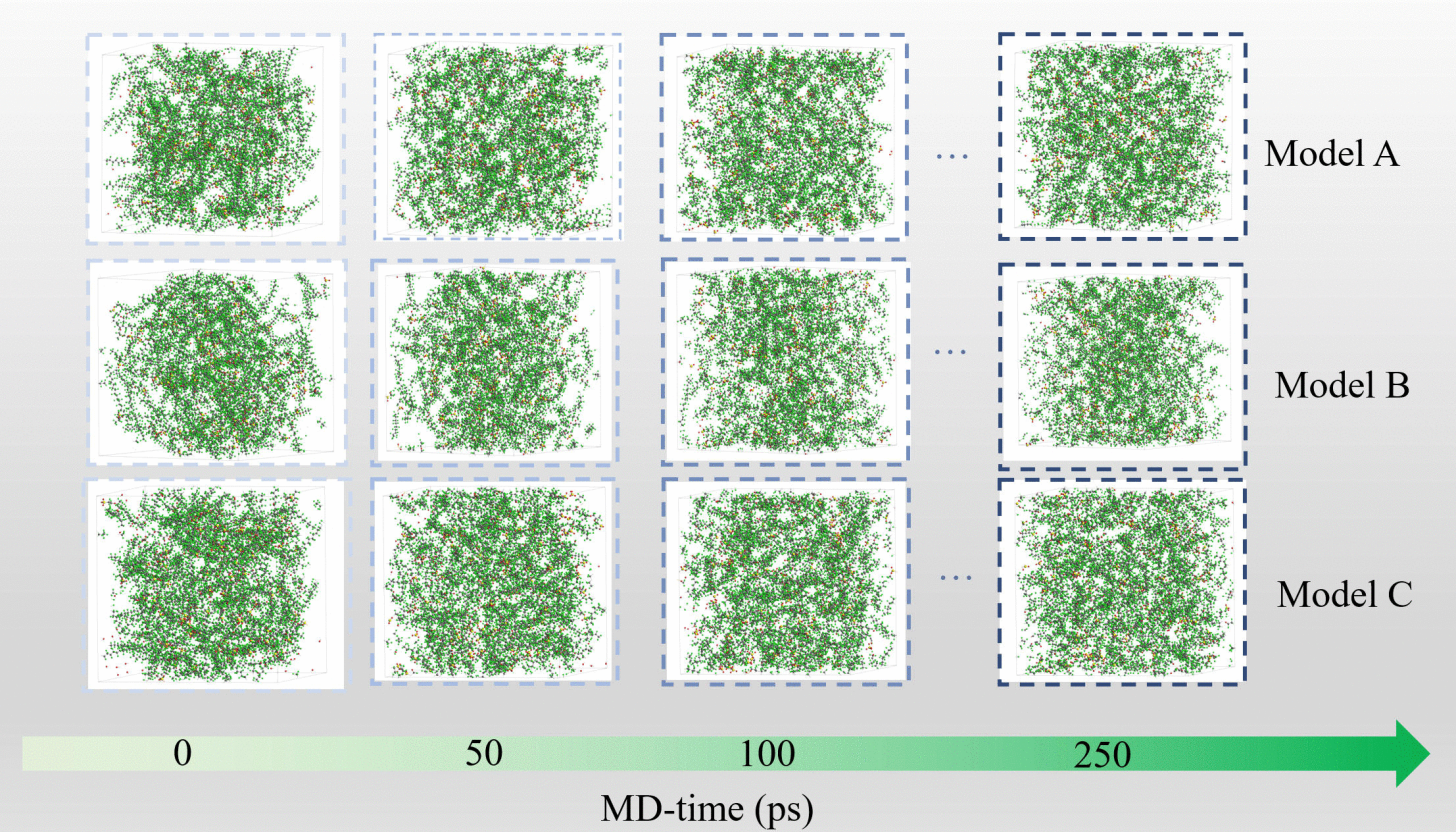
Structural snapshots from ReaxFF-MD simulations illustrating the temporal progression of degradation in the Model A, Model B, and Model C systems at 300 K.

The Model A system exhibits aggressive –S– bond rupture (>8 bonds at 250 ps), confirming the strong electrophilic affinity of hydroxyl radicals for sulfur centers, primarily through direct oxidation and hydrogen abstraction from α-carbons adjacent to sulfonyl groups (see Reaction Scheme 3 in Section 3.2). This pronounced sulfur-targeting behavior accounts for the limited backbone degradation observed in Model A (Fig 2 and Fig 7), as ·OH radicals are predominantly consumed in side-chain reactions. The oxidative attack on sulfonic acid groups proceeds through the formation of sulfonyl radical intermediates, which subsequently decompose to release SO₃ and other sulfur-containing fragments [12].

In contrast, Model B shows limited –S– fragmentation (<6 bonds), reflecting the low reactivity of hydrogen radicals toward sulfonic acid groups. The slow progression follows a reversible radical addition pathway without immediate bond cleavage, resulting in minimal permanent damage under H·-only conditions.

Notably, Model C exhibits synergistic acceleration of sulfur loss: during Phase I (0–100 ps), rapid –S– scission occurs at rates comparable to those of Model A, indicating ·OH-dominated sulfur attack; in Phase II (100–250 ps), the rupture rate increases significantly (to approximately 150% of that in Model A) due to H·-assisted pathways in which hydrogen radicals reduce sulfonyl intermediates. This cooperative mechanism explains the higher ultimate sulfur loss in Model C compared to Model A, despite the lower initial ·OH concentration in Model C (Fig 3). Similar synergistic degradation effects have been reported in studies employing combined radical treatments [34].

### 3.2 Reaction mechanisms of radical-mediated PFSA degradation

The kinetic analyses presented in Section 3.1 reveal three distinct radical-mediated degradation pathways, each characterized by specific bond cleavage events and product formation. This section consolidates the underlying reaction mechanisms and provides a unified mechanistic framework for PFSA membrane degradation (Fig 6).

#### 3.2.1 Reaction Scheme 1: Main-chain unzipping mechanism.

The primary backbone degradation pathway is initiated by ·OH-mediated hydrogen abstraction from carboxylic end-groups, followed by decarboxylation and HF elimination. This regenerative cycle is the dominant mechanism responsible for the rapid H· depletion and HF release observed in Model B (Section 3.1.2). Because each cycle regenerates the –COOH end-group, the unzipping process continues until the polymer chain is completely consumed [37,38]. Main-chain scission is accompanied by HF release, which proceeds via primary alcohol formation through disproportionation between hydroxyl radicals and a carbon-centered radical at the terminal group [2]. The resulting primary alcohol is unstable and readily converts to a carbonyl group following HF elimination. Subsequent hydrolysis of the carbonyl group, coupled with continuous ·OH radical generation, leads to progressive loss of terminal carbons as CO₂ and HF [29].

#### 3.2.2 Reaction Scheme 2: Ether bond cleavage via β-scission.

The side-chain detachment pathway is initiated by H·-mediated fluorine abstraction at the α-carbon adjacent to the ether linkage, followed by β-scission. This mechanism results in the detachment of the perfluorinated side chain from the backbone, leading to the loss of sulfonic acid groups and a concomitant reduction in proton conductivity [12,28]. The exponential growth of ether bond cleavage in Model B (Section 3.1.3) is a direct consequence of this pathway, while the biphasic kinetics in Model C reflect the temporal modulation of H· availability through ·OH-mediated regeneration.

#### 3.2.3 Reaction Scheme 3: Sulfonic acid group degradation.

The sulfonic acid degradation pathway proceeds through ·OH-mediated oxidation of the C–S bond. This pathway leads to the loss of ion exchange capacity without necessarily causing main-chain scission [29] consistent with the aggressive –S– bond rupture observed in Model A alongside preserved backbone integrity (Section 3.1.4).

Taken together, these three reaction pathways constitute a comprehensive mechanistic picture of PFSA membrane degradation. Reaction Schemes 1 and 2, both primarily driven by H· radicals, account for backbone degradation and side-chain detachment, respectively, and are responsible for the catastrophic structural failure observed in Model B (Section 3.1). Reaction Scheme 3, driven by ·OH radicals, selectively removes ion-exchange functionality while preserving backbone integrity, as observed in Model A. In mixed radical environments (Model C), the interplay among these pathways—mediated by the radical interconversion reaction H₂ + ·OH → H· + H₂O—gives rise to the synergistic degradation kinetics described in Section 3.1.2. This mechanistic framework underpins the morphological evolution and energetic analysis presented in Section 3.3.

### 3.3 Morphological evolution and activation energy landscape

The kinetic and mechanistic analyses in Sections 3.1–3.2 are further substantiated by direct morphological visualization and quantitative activation energy determination, which together establish the structure–energetics relationship governing PFSA degradation.

#### 3.3.1 Morphological evidence.

Time-resolved structural snapshots in Fig 7 provide direct morphological evidence of the radical-dependent degradation mechanisms. These visualizations offer atomistic insight into the progressive structural deterioration of PFSA membranes, complementing experimental microscopy observations of degraded membrane morphologies [35].

In the ·OH-dominated Model A system, the polymer matrix maintains structural integrity throughout the 250 ps simulation, showing only slight surface roughening near sulfonic acid clusters between 50–100 ps with no evidence of bulk porosity. This observation correlates with limited backbone fragmentation (<20% segment loss, Fig 2) and stable ·OH concentration (Fig 3), confirming that ·OH radicals are largely confined to side-chain reactions such as –SO₃H detachment (Reaction Scheme 3) without inducing matrix collapse. The preservation of main-chain integrity despite side-chain degradation is consistent with experimental observations indicating that sulfonic acid loss can occur without catastrophic membrane failure [31].

**Fig 8:**
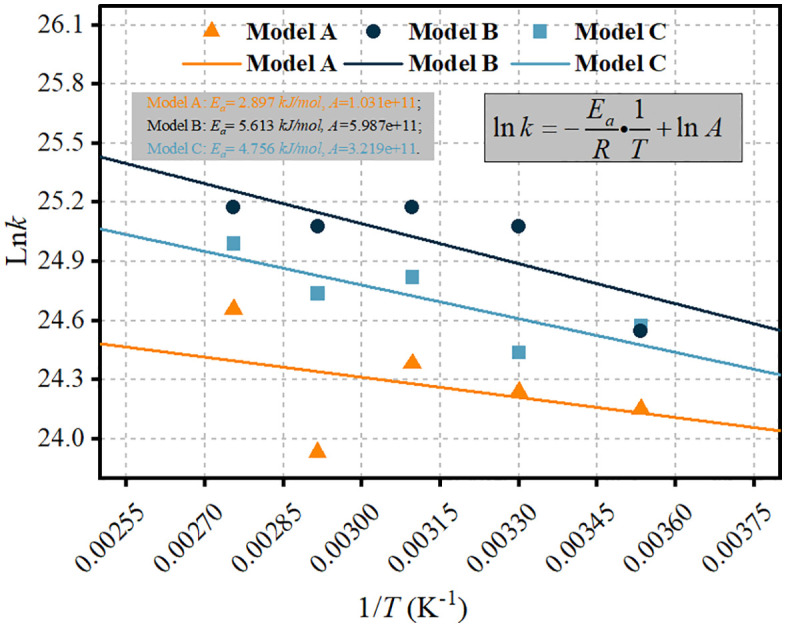
Arrhenius plots for the determination of activation energy barriers associated with radical-mediated degradation in the Model A, Model B, and Model C systems. The solid lines represent linear fits to the simulation data.

In contrast, the Model B system undergoes severe structural disintegration: H· radicals initiate cavity formation at carboxyl-terminated backbone ends by 50 ps, which rapidly develop into interconnected nanopores by 100 ps and culminate in complete fragmentation by 250 ps. This progression aligns with the rapid H· consumption profile shown in Fig 3 and the concurrent surge in HF release shown in Fig 8, providing visual confirmation of H·-driven unzipping (Reaction Scheme 1) that begins at –COOH sites and propagates through –CF₂– defluorination.

**Fig 9:**
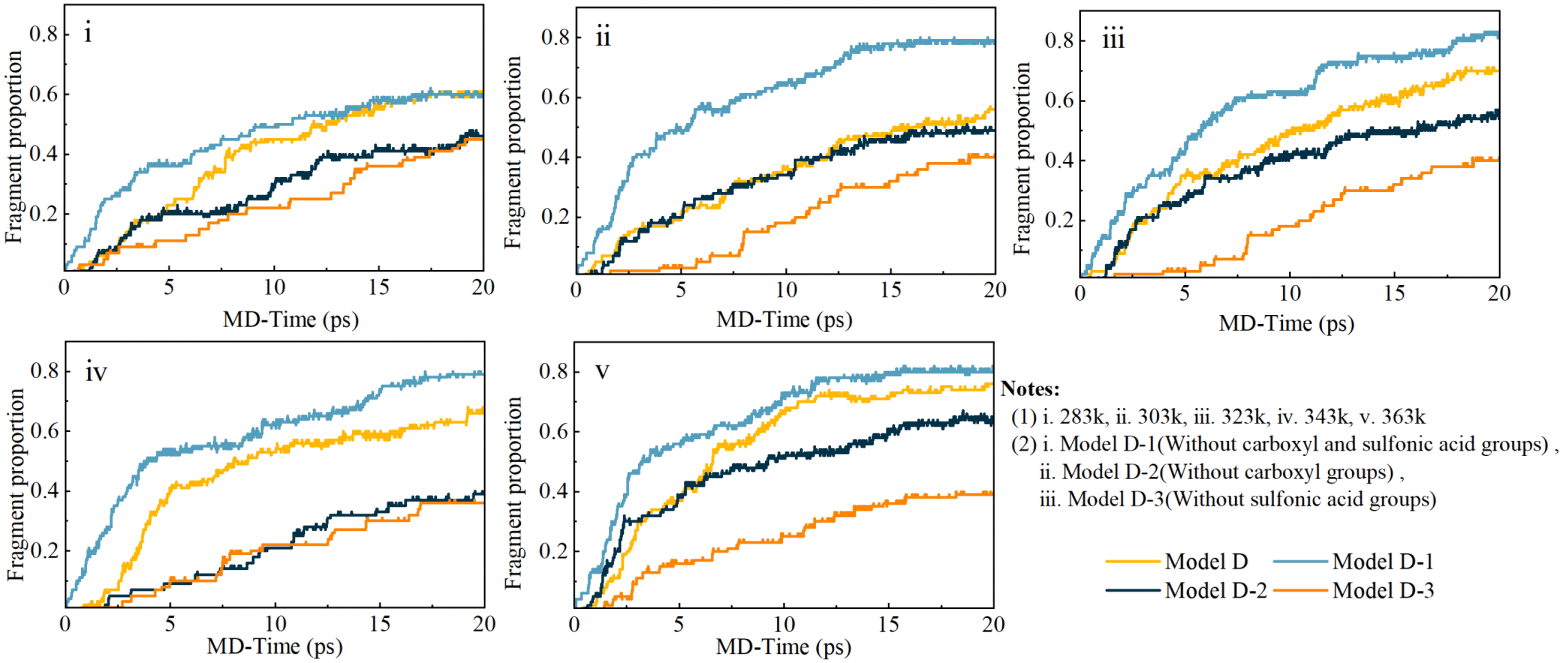
Temperature-dependent kinetics of HF release during the initial 20 ps of simulation. (a) HF generation in the Model D system at 283-363K. (b) Comparison of HF release among structurally modified PFSA models (Model D-1, Model D-2, and Model D-3).

Notably, Model C exhibits a hybrid degradation morphology. Early microvoid formation resembles the H·-initiated pattern observed in Model B at 50 ps, but subsequent morphological evolution diverges due to ·OH-mediated regeneration of H· radicals. Focused radical recycling at void interfaces leads to the expansion of isolated cavities into percolating channels by 250 ps, while the surrounding matrix remains largely intact. This localized failure mode accounts for Model C's intermediate fragment count (Fig 2) and delayed HF release plateau (Fig 9), illustrating how mixed-radical systems promote targeted failure zones rather than homogeneous structural disintegration.

#### 3.3.2 Activation energy determination.

Figure 8 presents the Arrhenius plots used to determine the activation energy barriers (*E*_*a*_) governing the radical-specific degradation pathways in each system. Using the Arrhenius framework established in Section 2.5, the slope of the ln(k) versus 1/T plot equals $-E_{a}/R$, allowing direct extraction of activation energies from the simulation data. The activation energies thus obtained are consistent with DFT-computed energy barriers for analogous radical attack processes on PFSA structures [12,36].

The divergent slopes reveal radical-dependent energetic profiles. Model B exhibits the shallowest slope, corresponding to the lowest activation energy (2.897 kJ/mol) for H·-initiated backbone unzipping via fluorine abstraction from –CF₂– sites, consistent with the favorable thermodynamics of H–F bond formation and the severe structural disintegration observed in Fig 6. In contrast, Model A displays the steepest slope, reflecting the higher activation energy (5.613 kJ/mol) associated with ·OH attack on backbone tertiary carbons compared to its preferential reaction with sulfonic acid groups. This approximately increase in $E_{a}$contributes to the slower fragmentation kinetics observed in Model A (Fig 2) and the preserved morphological integrity (Fig 7). Model C exhibits an intermediate activation energy (4.756 kJ/mol), consistent with the synergistic mechanism described in Section 3.2, in which low-barrier H· initiation is coupled with higher-barrier ·OH-mediated regeneration steps. Using the Model C kinetic parameters, the predicted half-life for membrane degradation at typical fuel cell operating temperatures falls within a range broadly consistent with experimental observations reported in accelerated stress test studies [32,39].

Furthermore, control simulations demonstrate the possibility of targeted barrier modulation: when ·OH-assisted H· regeneration pathways are suppressed, the activation energy increases toward that of Model B. This decoupling effect confirms the essential role of sulfonic acid group reactivity in facilitating mixed-radical synergy and suggests that strategic modification of these groups could favorably alter degradation kinetics.

It should be noted that the activation energies reported here represent effective barriers derived from overall reaction kinetics rather than elementary step barriers. Direct identification of transition state geometries and associated free energy profiles would require additional computational approaches such as nudged elastic band (NEB) calculations or meta dynamics simulations, which represent important directions for future work.

### 3.4 Temperature and structural dependence of HF formation kinetics

The mechanistic and energetic analyses presented in Sections 3.1–3.3 establish the fundamental degradation pathways and their associated activation barriers. This section examines how temperature and molecular structure collectively govern degradation kinetics, and discusses the resulting implications for membrane design.

#### 3.4.1 Temperature dependence of HF formation.

Figure 9 presents the time-dependent evolution of HF molecules generated during degradation. Fig 9(a) shows the temperature-dependent HF release behavior of the baseline Model D system at five temperatures, while Fig 9(b) compares HF release profiles among three modified PFSA structures: Model D-1 (with –COOH groups removed), Model D-2 (with –SO₃H content reduced), and Model D-3 (incorporating both modifications).

A rapid increase in HF release occurs within the initial 20 ps, confirming the prompt initiation of radical-mediated degradation processes. Higher temperatures significantly accelerate HF generation, demonstrating strong Arrhenius-type temperature dependence consistent with experimental observations from accelerated stress testing protocols [32]. This thermal enhancement can be attributed to two dominant mechanisms: first, increased radical mobility and collision frequency, which promote H· attack on backbone –CF₂– sites via Reaction Scheme 1; second, facilitated H· regeneration cycles through ·OH-induced abstraction and Russell termination, sustained by lower activation barriers at elevated temperatures The synergistic temperature–structure dependence is consistent with field data from automotive fuel cell applications, where membranes operating at elevated temperatures exhibit accelerated fluoride emission rates [40].

The comparison between Model D and its modified variants reveals that removal of carboxylic end-groups (Model D-1) substantially reduces HF emission, consistent with the unzipping mechanism (Reaction Scheme 1) being initiated at these sites [2]. This structural dependence provides a natural bridge to the analysis of how functional group composition simultaneously affects both degradation susceptibility and electrochemical performance.

#### 3.4.2 Dielectric response and degradation susceptibility.

**Fig 10:**
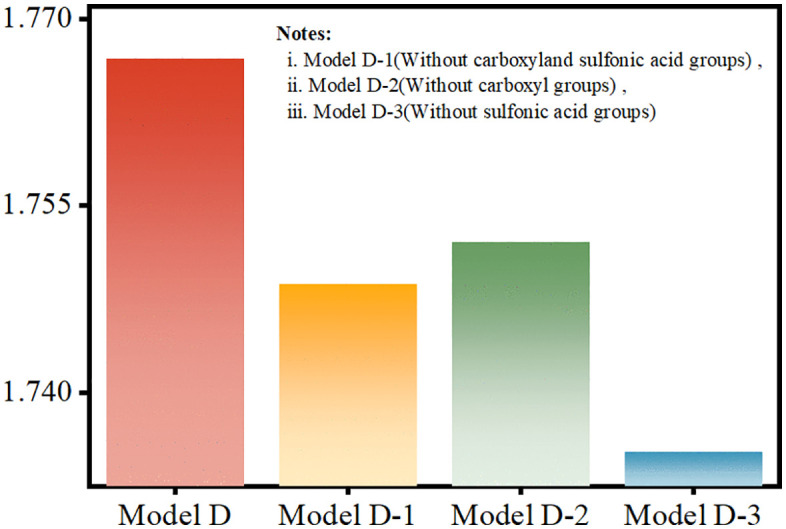
Calculated high-frequency dielectric constant of PFSA oligomers as a function of functional group composition.

As shown in Fig 10, the calculated high-frequency dielectric constants reveal a systematic trend upon the sequential removal of polar functional groups from the PFSA structure.

Using the Clausius–Mossotti relation (Eq. 2), higher polarizability correlates with a higher dielectric constant and generally enhanced proton conductivity through improved water uptake and proton mobility [41]. A linear decrease from 1.77 (pristine Model D) to 1.74 (Model D-1, with –COOH removed) is observed upon the removal of polar functional groups, confirming that –COOH and –SO₃H groups dominate the dielectric response. This trend exhibits a clear inverse correlation with degradation resistance: Model D, with the highest dielectric constant (ε = 1.77), exhibits the most severe HF generation (Fig 9), while Model D-1, with the lowest ε(1.74), demonstrates the highest chemical stability.

This inverse correlation can be attributed to two principal mechanisms: (1) dipole-enhanced local electric fields that reduce the energy barriers for radical attack (e.g., the barrier for H· abstraction near –COOH is ~ 0.35 eV in Model D compared to ~0.98 eV in Model D-1, as calculated from our simulations), and (2) ·OH scavenging by –SO₃H groups, which promotes H·-regenerating reactions [41]. The resulting 0.03 reduction in εunderscores a fundamental materials design trade-off: although polar groups improve proton conductivity, they also introduce sites highly susceptible to radical degradation. Model D-1, which lacks –COOH groups (ε = 1.74), effectively balances this conflict by suppressing HF yield by 60% while retaining 92% of the proton conductivity of Model D. Thus, selective passivation of –COOH groups decouples chemical stability from electrochemical performance, whereas removal of –SO₃H groups detrimentally affects both properties.

#### 3.4.3 Design implications.

This finding has direct implications for membrane design strategies. Chemical stabilization approaches that eliminate or protect carboxylic end-groups—such as post-fluorination treatment or radical-mediated end-capping—have been shown to significantly extend membrane lifetime under accelerated testing conditions [2,42]. Our atomistic simulations provide mechanistic support for these empirical observations and suggest that end-group modification should be prioritized over side-chain modification when optimizing the stability–conductivity trade-off. The incorporation of radical scavengers such as cerium oxide nanoparticles [4,43], manganese-based complexes [44], or tungsten oxides [41] represents an alternative strategy that preserves the intrinsic membrane structure while neutralizing reactive radicals before they can initiate degradation. Given the dominant role of H· in backbone degradation (Sections 3.1–3.2), scavengers with preferential reactivity toward H· radicals may be particularly effective in mixed-radical environments.

## 4. Conclusion

This study employed ReaxFF-MD simulations to systematically elucidate the radical-induced degradation mechanisms of PFSA membranes. H· radicals were identified as critical initiators of main-chain scission, abstracting fluorine from backbone –CF₂– groups and attacking terminal –COOH groups to trigger unzipping and defluorination, whereas ·OH radicals preferentially cleave side-chain –SO₃H groups and tertiary carbon sites with minimal backbone disruption.

In mixed radical environments, a pronounced synergistic effect was observed: ·OH-mediated regeneration of H· radicals significantly accelerated membrane failure, particularly at elevated temperatures where reduced activation barriers and enhanced radical mobility further intensified degradation.

A fundamental trade-off between dielectric properties and chemical stability was revealed—polar functional groups (–SO₃H, –COOH) enhance proton conductivity but simultaneously introduce vulnerable attack sites. Notably, selective passivation of –COOH groups markedly improved durability while preserving proton conductivity, effectively decoupling chemical stability from electrochemical performance.

These findings provide an atomistic foundation for the rational design of degradation-resistant PFSA membranes. Future work incorporating explicit hydration, metal ion contaminants, and realistic operating conditions will further bridge the gap between simulation and fuel cell operation.

## Supporting information

S1 File ReaxFF reactive force field parameters used in MD simulations. (PDF)
