# Supplementary material for: Atomistic insights into the degradation of perfluorosulfonic acid membranes: A reactive force field molecular dynamics study
Source: PLoS One. 2026 Apr 7;21(4):e0346636. doi: 10.1371/journal.pone.0346636 (PMC13056174; doi:10.1371/journal.pone.0346636)
Supplement: S1 File — (PDF) [file pone.0346636.s001.pdf]

The reactive force field employed in this work was constructed by building upon a series of previously validated ReaxFF parameterizations. The core C/H/O/N/S/Mg/P/Na/Li parameters were taken from Bedrov et al. (2012), who developed and validated this parameter set for simulating reduction reactions of ethylene carbonate in lithium battery electrolytes using ReaxFF. The B/F interaction parameters were subsequently incorporated from Islam, Bryantsev, and van Duin (2014), derived from ReaxFF simulations of Teflon-containing Li–S battery systems. The complete force field was then recast within the eReaxFF/ACKS2 framework by Islam and van Duin (2016), which extends the standard ReaxFF methodology to treat explicit electron transfer via pseudo-particles under the ACKS2 charge equilibration scheme.

The present work adopts this force field the published parameters, retaining the ACKS2 charge equilibration environment consistent with the original parameterization. This ensures methodological continuity with the subsequent eReaxFF simulations planned for the PEMFC cathode system, in which explicit electron and hole pseudo-particles will be activated.

References:

1 Islam, M.; van Duin, A. C. T. J. Phys. Chem. C 2016, 120, 27128–27134. https://doi.org/10.1021/acs.jpcc.6b08688

2 Islam, M. M.; Bryantsev, V. S.; van Duin, A. C. T. J. Electrochem. Soc. 2014, 161, E3009–E3014. https://doi.org/10.1149/2.005408jes

3 Bedrov, D.; Smith, G. D.; van Duin, A. C. T. J. Phys. Chem. A 2012, 116, 2978–2985. https://doi.org/10.1021/jp210345b

```
41       ! Number of general parameters
  50.0000 !Overcoordination parameter
   9.5469 !Overcoordination parameter
   1.6725 !Valency angle conjugation parameter
   1.7224 !Triple bond stabilisation parameter
   6.8702 !Triple bond stabilisation parameter
  54.6742 !C2-correction
   1.0588 !Undercoordination parameter
   4.6000 !Triple bond stabilisation parameter
  12.1176 !Undercoordination parameter
  13.3056 !Undercoordination parameter
 -34.5448 !Triple bond stabilization energy
   0.0000 !Lower Taper-radius
  10.0000 !Upper Taper-radius
   2.8793 !Not used
  33.8667 !Valency undercoordination
   6.0891 !Valency angle/lone pair parameter
   1.0563 !Valency angle
   2.0384 !Valency angle parameter
   6.1431 !Not used
   6.9290 !Double bond/angle parameter
   0.3989 !Double bond/angle parameter: overcoord
   3.9954 !Double bond/angle parameter: overcoord
```

```
  -2.4837 !Not used
   8.5385 !Torsion/BO parameter
   6.7491 !Torsion overcoordination
   0.1414 !Torsion overcoordination
   1.0000 !Conjugation 0 (not used)
   1.1348 !Conjugation
   1.5591 !vdWaals shielding
   0.1000 !Cutoff for bond order (*100)
   1.7602 !Valency angle conjugation parameter
   0.6991 !Overcoordination parameter
  50.0000 !Overcoordination parameter
   1.8512 !Valency/lone pair parameter
 548.6451 !Softness
   0.0001 !nu
   1.5000 !Pval
  10.0000 !Upper taper radius for e-reax
   0.7903 !Valency angle conjugation parameter
   0.0000 !n u
   5.0000 ! electron taper radius

15    ! Nr of atoms; cov.r; valency;a.m;Rvdw;Evdw;gammaEEM;cov.r2;#
            alfa;gammavdW;valency;Eunder;Eover;chiEEM;etaEEM;n.u.
            cov r3;Elp;Heat inc.;n.u.;n.u.;n.u.;n.u.
            ov/un;val1;n.u.;val3,vval4
C    1.3825   4.0000  12.0000   1.9133   0.1853   0.9000   1.1359   4.0000
     9.7602   2.1346   4.0000  33.2433  79.5548   5.8678   7.0000   0.0000
     1.2104   0.0000 199.0303   8.6991  34.7289  13.3894   0.8563   0.0000
    -2.8983   2.5000   1.0564   4.0000   2.9663   0.0000   0.0000   0.0000
H    0.7853   1.0000   1.0080   1.5904   0.0419   1.0206  -0.1000   1.0000
     9.3557   5.0518   1.0000   0.0000 121.1250   5.3200   7.4366   1.0000
    -0.1000   0.0000  62.4879   1.9771   3.3517   0.7571   1.0698   0.0000
   -15.7683   2.1488   1.0338   1.0000   2.8793   0.0000   0.0000   0.0000
O    1.2477   2.0000  15.9990   1.9236   0.0904   1.0503   1.0863   6.0000
    10.2127   7.7719   4.0000  36.9573 116.0768   8.5000   8.9989   2.0000
     0.9088   1.0003  60.8726  20.4140   3.3754   0.2702   0.9745   0.0000
    -3.6141   2.7025   1.0493   4.0000   2.9225   0.0000   0.0000   0.0000
N    1.2333   3.0000  14.0000   1.9324   0.1376   0.7921   1.1748   5.0000
    10.0667   7.8431   4.0000  32.2482   0.8000   7.5795   6.3952   3.0879
     1.0433  27.4290 119.9837   1.9457   4.2874   3.4869   1.2000   0.0000
    -4.3875   2.6192  -0.0673   4.0000   2.8793   0.0000   0.0000   0.0000
S    1.9405   6.0000  32.0600   2.0677   0.2099   1.0336   1.5479   6.0000
     9.9575   4.9055   4.0000  52.9998 112.1416   6.5000   8.2545   2.0000
     1.4601   9.7177  71.1843   5.7487  23.2859  12.7147   0.9745   0.0000
   -11.0000   2.7466   1.0338   6.2998   2.8793   0.0000   0.0000   0.0000
Mg   1.8315   2.0000  24.3050   2.2464   0.1806   0.5020   1.0000   2.0000
    10.9186  27.1205   3.0000  38.0000   0.0000   0.9499   5.6130   0.0000
    -1.3000   0.0000 220.0000  49.9248   0.3370   0.0000   0.0000   0.0000
    -1.0823   2.3663   1.0564   6.0000   2.9663   0.0000   0.0000   0.0000
```

```
P    1.5994   3.0000  30.9738   1.7000   0.1743   1.0000   1.3000   5.0000
     9.1909  14.9482   5.0000   0.0000   0.4000   1.6676   7.0946   4.0428
    -1.0000  25.0000   1.5000   0.2187  21.4305  15.1425   4.0433   0.0000
    -3.9294   3.4831   0.0210   5.0000   2.8793   0.0000   0.0000   0.0000
Na   2.0300   1.0000  22.9898   2.3334   0.1481   0.8765  -1.0000   1.0000
    11.0000   9.8000   1.0000   0.0000   0.0000  -3.8501   5.9459   0.0000
    -1.0000   0.0000  67.5458 100.0000  10.0000   0.2500   0.8563   0.0000
    -2.5766   2.5000   1.0338   6.0000   2.5791   0.0000   0.0000   0.0000
F    1.2100   1.0000  18.9984   1.8601   0.1200   0.3000  -0.1000   7.0000
    11.5000   7.5000   4.0000   9.2533   0.2000   9.0000  15.0000   0.0000
    -1.0000  35.0000   1.5000   6.9821   4.1799   1.0561   0.0000   0.0000
    -7.3000   2.6656   1.0493   4.0000   2.9225   0.0000   0.0000   0.0000
B    1.1936   3.0000  10.8110   1.8368   0.0708   0.7695  -1.0000   3.0000
     9.0332   3.6413   3.0000  38.0000   0.4000   2.3652   8.5128   3.0925
    -1.3000   0.0000 220.0000   5.4000  24.0000  12.0000  10.1764   0.0000
   -10.8644   2.4601   0.0028   6.2998   2.9663   0.0000   0.0000   0.0000
Li   1.9814   1.0000   6.9410   1.8000   0.2939   0.9445  -0.1000   1.0000
     9.0616   1.3258   1.0000   0.0000   0.2000  -3.0000  10.0241   6.0000
    -1.0000   0.0000  37.5000   5.4409   6.9107   0.1973   0.8563   0.0000
    -2.5068   2.2989   0.0138   1.0000   2.8103   0.0000   0.0000   0.0000
Lv   1.9814   0.0000   6.9410   1.8000   0.2939   0.4839  -0.1000   0.0000
     9.0616   1.3258   1.0000   0.0000   0.2000  -1.2781  10.0339   5.4886
    -1.0000   0.0000  37.5000   5.4409   6.9107   0.1973   5.4886   1.0000
    -2.0000   2.2989   0.0020   1.0000   2.8103   0.0000   0.0000   0.0000
El  -0.1000   0.0000   1.0000   1.3000   0.0050   0.2253  -0.1000   0.0000
     2.0000   4.0000   4.0000   0.0000   0.0000   0.9882   1.0139  10.0000
    -0.1000   0.0000  -2.3700   6.4918   8.5961   0.2368   2.0000   0.0000
    -5.0000   3.1873   1.0338   6.2998   2.5791   0.0000   0.0000   0.0000
Ho  -0.1000   0.0000   1.0000   1.3000   0.0050   0.3514  -0.1000   0.0000
     1.2827   4.0000   1.0000   0.0000   0.0000   3.5706   3.5741  10.0000
    -0.1000   0.0000  -2.3700   0.0018   8.5961   0.2368  10.0000   2.0000
    -5.0000   0.0073   0.0338   0.0098   2.5791   0.0000   0.0000   0.0000
X   -0.1000   2.0000   1.0080   2.0000   0.0000   1.0000  -0.1000   6.0000
    10.0000   2.5000   4.0000   0.0000   0.0000   8.5000   1.5000  10.0000
    -0.1000   0.0000  -2.3700   8.7410  13.3640   0.6690   0.9745   0.0000
   -11.0000   2.7466   1.0338   4.0000   2.8793   0.0000   0.0000   0.0000
60    ! Nr of bonds; Edis1;LPpen;n.u.;pbe1;pbo5;13corr;pbo6
                       pbe2;pbo3;pbo4;Etrip;pbo1;pbo2;ovcorr
  1   1 100.5953 100.0397  80.0000  -0.8157  -0.4591   1.0000  37.7369   0.4235
         0.4527  -0.1000   9.2605   1.0000  -0.0750   6.8316   1.0000   0.0000
  1   2 170.2316   0.0000   0.0000  -0.5931   0.0000   1.0000   6.0000   0.7140
         5.2267   1.0000   0.0000   1.0000  -0.0500   6.8315   0.0000   0.0000
  2   2 156.0973   0.0000   0.0000  -0.1377   0.0000   1.0000   6.0000   0.8240
         2.9907   1.0000   0.0000   1.0000  -0.0593   4.8358   0.0000   0.0000
  1   3 160.4802 105.1693  23.3059  -0.3873  -0.1613   1.0000  10.8851   1.0000
         0.5341  -0.3174   7.0303   1.0000  -0.1463   5.2913   0.0000   0.0000
  3   3  60.1463 176.6202  51.1430  -0.2802  -0.1244   1.0000  29.6439   0.9114
         0.2441  -0.1239   7.6487   1.0000  -0.1302   6.2919   1.0000   0.0000
```

2	3	180.4373	0.0000	0.0000	-0.8074	0.0000	1.0000	6.0000	0.5514
		1.2490	1.0000	0.0000	1.0000	-0.0657	5.0451	0.0000	0.0000
1	4	134.1215	140.2179	79.9745	0.0163	-0.1428	1.0000	27.0617	0.2000
		0.1387	-0.3681	7.1611	1.0000	-0.1000	5.0825	1.0000	0.4000
3	4	130.8596	169.4551	40.0000	0.3837	-0.1639	1.0000	35.0000	0.2000
		1.0000	-0.3579	7.0004	1.0000	-0.1193	6.8773	1.0000	0.2000
4	4	157.9384	82.5526	152.5336	0.4010	-0.1034	1.0000	12.4261	0.5828
		0.1578	-0.1509	11.9186	1.0000	-0.0861	5.4271	1.0000	0.4000
2	4	185.3171	0.0000	0.0000	-0.3689	0.0000	1.0000	6.0000	0.2854
		7.6517	1.0000	0.0000	1.0000	-0.0408	6.0255	0.0000	0.5000
1	5	128.9942	74.5848	55.2528	0.1035	-0.5211	1.0000	18.9617	0.6000
		0.2949	-0.2398	8.1175	1.0000	-0.1029	5.6731	1.0000	0.0000
2	5	151.5159	0.0000	0.0000	-0.4721	0.0000	1.0000	6.0000	0.6000
		9.4366	1.0000	0.0000	1.0000	-0.0290	7.0050	1.0000	0.0000
3	5	107.2917	202.9813	40.0000	0.4728	-0.2406	1.0000	22.1005	0.0500
		0.6528	-0.3341	7.9877	1.0000	-0.0909	6.9512	1.0000	0.0000
4	5	0.0000	0.0000	0.0000	0.4438	-0.2034	1.0000	40.3399	0.6000
		0.3296	-0.3153	9.1227	1.0000	-0.1805	5.6864	1.0000	0.0000
5	5	96.1871	93.7006	68.6860	0.0955	-0.4781	1.0000	17.8574	0.6000
		0.2723	-0.2373	9.7875	1.0000	-0.0950	6.4757	1.0000	0.0000
2	6	58.6896	0.0000	0.0000	-0.0203	-0.1418	1.0000	13.1260	0.0230
		8.2136	-0.1310	0.0000	1.0000	-0.2692	6.4254	0.0000	24.4461
3	6	87.0227	0.0000	43.3991	0.0030	-0.3000	1.0000	36.0000	0.0250
		0.0087	-0.2500	12.0000	1.0000	-0.0439	6.6073	1.0000	24.4461
6	6	32.3808	0.0000	0.0000	-0.0076	-0.2000	0.0000	16.0000	0.2641
		4.8726	-0.2000	10.0000	1.0000	-0.0729	4.6319	0.0000	0.0000
1	7	110.0000	92.0000	0.0000	0.2171	-0.1418	1.0000	13.1260	0.6000
		0.3601	-0.1310	10.7257	1.0000	-0.0869	5.3302	1.0000	0.0000
2	7	0.1466	0.0000	0.0000	0.2250	-0.1418	1.0000	13.1260	0.6000
		0.3912	-0.1310	0.0000	1.0000	-0.1029	9.3302	0.0000	0.0000
3	7	201.0058	194.1410	0.0000	1.0000	-0.5000	1.0000	25.0000	0.4873
		0.4358	-0.1571	15.8745	1.0000	-0.2431	6.3823	1.0000	0.0000
4	7	130.0000	0.0000	0.0000	0.2171	-0.1418	1.0000	13.1260	0.6000
		0.3601	-0.1310	10.7257	1.0000	-0.0869	5.3302	1.0000	0.0000
6	7	0.1000	0.0000	0.0000	0.2500	-0.5000	1.0000	35.0000	0.6000
		0.5000	-0.5000	20.0000	1.0000	-0.2000	10.0000	1.0000	0.0000
7	7	0.0000	0.0000	0.0000	0.2171	-0.5000	1.0000	35.0000	0.6000
		0.5000	-0.5000	20.0000	1.0000	-0.2000	10.0000	1.0000	0.0000
2	8	0.0000	0.0000	0.0000	-1.0000	-0.3000	1.0000	36.0000	0.7000
		10.1151	-0.3500	25.0000	1.0000	-0.1053	8.2003	1.0000	0.0000
3	8	76.0753	0.0000	0.0000	-0.4452	-0.3000	1.0000	36.0000	0.6433
		5.6834	-0.3500	25.0000	1.0000	-0.0539	8.0273	1.0000	0.0000
4	8	0.0000	0.0000	0.0000	-1.0000	-0.3000	1.0000	36.0000	0.7000
		10.1151	-0.3500	25.0000	1.0000	-0.1053	8.2003	1.0000	0.0000
6	8	0.1000	0.0000	0.0000	0.2500	-0.5000	1.0000	35.0000	0.6000
		0.5000	-0.5000	20.0000	1.0000	-0.2000	10.0000	1.0000	0.0000
7	8	0.1000	0.0000	0.0000	0.2500	-0.5000	1.0000	35.0000	0.6000
		0.5000	-0.5000	20.0000	1.0000	-0.2000	10.0000	1.0000	0.0000

8	8	27.8052	0.0000	0.0000	0.4022	0.3000	0.0000	25.0000	0.4894
		0.6222	-0.4000	12.0000	1.0000	-0.0500	5.3362	0.0000	0.0000
4	6	0.0000	0.0000	0.0000	-1.0000	-0.3000	1.0000	36.0000	0.7000
		10.1151	-0.3500	25.0000	1.0000	-0.1053	8.2003	1.0000	0.0000
1	9	237.8781	0.0000	0.0000	-0.7438	-0.5000	1.0000	35.0000	1.0460
		3.6661	-0.2500	15.0000	1.0000	-0.0800	5.4719	1.0000	0.0000
2	9	0.0000	0.0000	0.0000	-0.4643	0.0000	1.0000	6.0000	0.6151
		12.3710	1.0000	0.0000	1.0000	-0.1008	8.5980	0.0000	0.0000
3	9	0.0000	0.0000	0.0000	0.2500	-0.5000	1.0000	45.0000	0.6000
		0.4000	-0.2500	15.0000	1.0000	-0.1000	10.0000	1.0000	0.0000
4	9	0.0000	0.0000	0.0000	-0.4643	0.0000	1.0000	6.0000	0.6151
		12.3710	1.0000	0.0000	1.0000	-0.0098	8.5980	0.0000	0.0000
5	9	0.0000	0.0000	0.0000	-0.4643	0.0000	1.0000	6.0000	0.6151
		12.3710	1.0000	0.0000	1.0000	-0.0098	8.5980	0.0000	0.0000
7	9	182.8277	0.0000	0.0000	-0.2394	-0.5000	1.0000	50.0000	0.1000
		0.2419	-0.5000	15.0000	1.0000	-0.0913	4.9081	1.0000	0.4000
9	9	250.0765	0.0000	0.0000	0.2298	-0.3500	1.0000	25.0000	0.8427
		0.1167	-0.2500	15.0000	1.0000	-0.1506	7.3516	1.0000	0.0000
1	10	0.0000	0.0000	0.0000	0.2500	-0.5000	1.0000	45.0000	0.7500
		0.5000	-0.4000	20.0000	1.0000	-0.2000	9.0000	1.0000	0.0000
2	10	0.0000	0.0000	0.0000	0.2500	-0.5000	1.0000	45.0000	0.7500
		0.5000	-0.4000	20.0000	1.0000	-0.2000	9.0000	1.0000	0.0000
3	10	0.0000	0.0000	0.0000	0.2500	-0.5000	1.0000	45.0000	0.7500
		0.5000	-0.4000	20.0000	1.0000	-0.2000	9.0000	1.0000	0.0000
4	10	0.0000	0.0000	0.0000	0.2500	-0.5000	1.0000	45.0000	0.7500
		0.5000	-0.4000	20.0000	1.0000	-0.2000	9.0000	1.0000	0.0000
9	10	151.5296	0.0000	0.0000	-2.4475	-0.5000	1.0000	50.0000	0.1940
		0.0084	-0.2500	40.0000	1.0000	-0.1919	4.5000	1.0000	0.5000
10	10	0.0000	0.0000	0.0000	0.2500	-0.5000	1.0000	45.0000	0.7500
		0.5000	-0.4000	20.0000	1.0000	-0.2000	9.0000	1.0000	0.0000
1	11	54.6610	-0.0200	0.0000	-0.8605	-0.5000	0.0000	35.0000	0.3953
		0.6908	-0.2500	11.9965	1.0000	-0.0668	9.0596	0.0000	0.0000
2	11	59.2034	0.0000	0.0000	0.1240	0.0000	0.0000	6.0000	0.4000
		1.0000	0.0000	12.0000	1.0000	-0.0565	4.9575	0.0000	0.0000
3	11	91.3670	-0.0200	0.0000	-0.2977	0.3000	0.0000	6.0000	0.4665
		1.5185	-0.2500	11.9965	1.0000	-0.0861	7.0486	0.0000	0.0000
4	11	0.0000	0.0000	0.0000	0.2500	-0.5000	1.0000	45.0000	0.6000
		0.4000	-0.2500	15.0000	1.0000	-0.1000	10.0000	1.0000	0.0000
11	11	34.3154	0.0000	0.0000	0.5995	0.3000	0.0000	26.0000	0.5445
		0.5752	0.0000	12.0000	1.0000	-0.1382	4.5000	0.0000	0.0000
9	11	127.9679	0.0000	0.0000	-0.9999	-0.5000	0.0000	45.0000	0.3649
		1.1131	-0.2500	15.0000	1.0000	-0.0700	4.5981	0.0000	0.0000
7	11	0.0000	0.0000	0.0000	0.2500	-0.5000	1.0000	45.0000	0.6000
		0.4000	-0.2500	15.0000	1.0000	-0.1000	10.0000	1.0000	0.0000
10	11	0.0000	0.0000	0.0000	0.2500	-0.5000	1.0000	45.0000	0.6000
		0.4000	-0.2500	15.0000	1.0000	-0.1000	10.0000	1.0000	0.0000
1	12	15.0000	-0.0200	0.0000	-1.0000	-0.5000	0.0000	35.0000	0.2500
		1.0000	-0.2500	11.9965	1.0000	-0.0500	6.6794	0.0000	0.1000

```
  2 12  59.2034   0.0000   0.0000   0.1240   0.0000   0.0000   6.0000   0.4000
         1.0000   0.0000  12.0000   1.0000  -0.0565   4.9575   0.0000   1.5000
  3 12  43.6414  -0.0200   0.0000   1.0000   0.3000   0.0000   6.0000   0.4000
         5.7114  -0.2500  11.9965   1.0000  -0.1800   4.4514   0.0000   1.0000
  4 12   0.0000   0.0000   0.0000   0.2500  -0.5000   1.0000  45.0000   0.6000
         0.4000  -0.2500  15.0000   1.0000  -0.1000  10.0000   1.0000   1.0603
 12 12  20.9578   0.0000   0.0000   0.9615   0.3000   0.0000  26.0000   1.0912
         0.9221   0.0000  12.0000   1.0000  -0.0524   5.0002   0.0000   0.1000
  9 12 127.9679   0.0000   0.0000  -0.9999  -0.5000   0.0000  45.0000   0.3649
         1.1131  -0.2500  15.0000   1.0000  -0.0700   4.5981   0.0000   0.4000
  7 12   0.0000   0.0000   0.0000   0.2500  -0.5000   1.0000  45.0000   0.6000
         0.4000  -0.2500  15.0000   1.0000  -0.1000  10.0000   1.0000   0.4000
 10 12   0.0000   0.0000   0.0000   0.2500  -0.5000   1.0000  45.0000   0.6000
         0.4000  -0.2500  15.0000   1.0000  -0.1000  10.0000   1.0000   0.4000
 27    ! Nr of off-diagonal terms; Ediss;Ro;gamma;rsigma;rpi;rpi2
  1  2   0.1219   1.4000   9.8442   1.1203  -1.0000  -1.0000
  2  3   0.0344   1.6800  10.3247   0.9013  -1.0000  -1.0000
  2  4   0.0687   1.5130  10.0094   0.9412  -1.0000  -1.0000
  1  3   0.1131   1.8523   9.8442   1.2775   1.1342   1.0621
  1  4   0.1447   1.8766   9.7990   1.3436   1.1885   1.1363
  3  4   0.1048   2.0003  10.1220   1.3173   1.1096   1.0206
  1  5   0.1408   1.8161   9.9393   1.7986   1.3021   1.4031
  2  5   0.0895   1.6239  10.0104   1.4640  -1.0000  -1.0000
  3  5   0.2832   1.8196  10.2295   1.4502   1.4557  -1.0000
  4  5   0.1505   1.9000  10.5104   1.8000   1.4000  -1.0000
  2  6   0.0100   1.6000  13.2979   1.8670  -1.0000  -1.0000
  3  6   0.0809   1.7000  11.4606   1.5177  -1.0000  -1.0000
  3  7   0.0534   1.7520  10.4281   1.8000   1.4498  -1.0000
  6  7   0.1801   1.8566   9.8498   0.1000  -1.0000  -1.0000
  3  8   0.1592   1.8283  11.7256   1.6655  -1.0000  -1.0000
  1  9   0.1254   1.6238  10.9942   1.3203  -1.0000  -1.0000
  2  9   0.0789   1.7577  10.5737   0.5586  -1.0000  -1.0000
  7  9   0.2016   1.8181   9.8289   1.3836  -1.0000  -1.0000
  9 10   0.0750   1.7484  10.2905   1.2951  -1.0000  -1.0000
  1 11   0.0270   2.4124  11.4640   1.7840   1.0000   1.0000
  2 11   0.1149   1.4658  11.0886   1.3337  -1.0000  -1.0000
  3 11   0.0741   1.6791  11.6248   1.8268   1.0000   1.0000
  9 11   0.1335   1.8000   9.7770   1.6126  -1.0000  -1.0000
  1 12   0.0248   1.0000  13.0000   1.8000   1.0000   1.0000
  2 12   0.1149   1.4658  11.0886   1.3337  -1.0000  -1.0000
  3 12   0.0234   1.0048   9.3311   1.4000   1.0000   1.0000
  9 12   0.1335   1.8000   9.7770   1.6126  -1.0000  -1.0000
101    ! Nr of angles;at1;at2;at3;Thetao,o;ka;kb;pv1;pv2
  1  1  1  67.2326  22.0695   1.6286   0.0000   1.7959  15.4141   1.8089
  1  1  2  65.2527  14.3185   6.2977   0.0000   0.5645   0.0000   1.1530
  2  1  2  70.0840  25.3540   3.4508   0.0000   0.0050   0.0000   3.0000
  1  2  2   0.0000   0.0000   6.0000   0.0000   0.0000   0.0000   1.0400
  1  2  1   0.0000   3.4110   7.7350   0.0000   0.0000   0.0000   1.0400
```

2	2	2	0.0000	27.9213	5.8635	0.0000	0.0000	0.0000	1.0400
1	1	3	49.5561	7.3771	4.9568	0.0000	0.7533	15.9906	1.0010
3	1	3	77.1171	39.8746	2.5403	-24.3902	1.7740	-42.9758	2.1240
1	1	4	78.5538	21.4381	7.4715	0.0000	1.1046	50.0000	1.5275
3	1	4	73.9544	12.4661	7.0000	0.0000	1.1046	0.0000	1.1880
4	1	4	89.3168	20.2660	7.5000	0.0000	1.1046	0.0000	1.5403
2	1	3	65.0000	14.2057	4.8649	0.0000	0.3504	0.0000	1.7185
2	1	4	74.2929	31.0883	2.6184	0.0000	0.1000	0.0000	1.0500
1	2	4	0.0000	0.0019	6.3000	0.0000	0.0000	0.0000	1.0400
1	3	1	74.3994	44.7500	0.7982	0.0000	3.0000	0.0000	1.0528
1	3	3	77.9854	36.6201	2.0201	0.0000	0.7434	67.0264	3.0000
1	3	4	82.4890	31.4554	0.9953	0.0000	3.0000	0.0000	1.0783
3	3	3	80.7324	30.4554	0.9953	0.0000	1.6310	50.0000	1.0783
3	3	4	84.3637	31.4554	0.9953	0.0000	3.0000	0.0000	1.0783
4	3	4	89.7071	31.4554	0.9953	0.0000	3.0000	0.0000	1.1519
1	3	2	71.5018	21.7062	0.4735	0.0000	0.5186	0.0000	1.1793
2	3	3	84.9468	23.3540	1.5057	0.0000	2.6374	0.0000	1.3023
2	3	4	75.6201	18.7919	0.9833	0.0000	0.1000	0.0000	1.0500
2	3	2	77.0645	10.4737	1.2895	0.0000	0.9924	0.0000	1.1043
1	4	1	81.4699	7.2318	1.2608	0.0000	3.0000	0.0000	1.2127
1	4	3	103.3204	33.0381	0.5787	0.0000	3.0000	0.0000	1.2127
1	4	4	50.0000	25.0250	4.7651	0.0000	3.0000	0.0000	1.2028
3	4	3	74.1978	42.1786	1.7845	-18.0069	3.0000	0.0000	1.2127
3	4	4	74.8600	43.7354	1.1572	-0.9193	3.0000	0.0000	1.2127
4	4	4	75.0538	14.8267	5.2794	0.0000	3.0000	0.0000	1.2127
1	4	2	68.2294	29.6576	1.0533	0.0000	0.3481	0.0000	1.5443
2	4	3	81.3686	40.0712	2.2396	0.0000	0.3481	0.0000	1.5443
2	4	4	83.0104	43.4766	1.5328	0.0000	0.3481	0.0000	1.5443
2	4	2	79.6336	17.7917	3.7832	0.0000	0.0222	0.0000	2.0238
1	2	3	0.0000	25.0000	3.0000	0.0000	1.0000	0.0000	1.0400
1	2	4	0.0000	0.0019	6.0000	0.0000	0.0000	0.0000	1.0400
1	2	5	0.0000	0.0019	6.0000	0.0000	0.0000	0.0000	1.0400
3	2	3	0.0000	0.0148	6.0000	0.0000	0.0000	0.0000	1.0400
3	2	4	0.0000	0.0019	6.0000	0.0000	0.0000	0.0000	1.0400
4	2	4	0.0000	0.0019	6.0000	0.0000	0.0000	0.0000	1.0400
2	2	3	0.0000	9.7025	6.0000	0.0000	0.0000	0.0000	1.0400
2	2	4	0.0000	0.0019	6.0000	0.0000	0.0000	0.0000	1.0400
1	1	5	74.4180	33.4273	1.7018	0.1463	0.5000	0.0000	1.6178
1	5	1	79.7037	28.2036	1.7073	0.1463	0.5000	0.0000	1.6453
2	1	5	63.3289	29.4225	2.1326	0.0000	0.5000	0.0000	3.0000
1	5	2	85.9449	38.3109	1.2492	0.0000	0.5000	0.0000	1.1000
1	5	5	85.6645	40.0000	2.9274	0.1463	0.5000	0.0000	1.3830
2	5	2	83.8555	5.1317	0.4377	0.0000	0.5000	0.0000	3.0000
2	5	5	97.0064	32.1121	2.0242	0.0000	0.5000	0.0000	2.8568
2	2	5	0.0000	0.0019	6.0000	0.0000	0.0000	0.0000	1.0400
5	4	5	62.0000	33.4273	1.7018	0.1463	0.5000	0.0000	1.0500
3	5	3	81.0926	30.2268	6.4132	-5.4471	2.5968	0.0000	3.0000
1	5	3	70.0000	35.0000	3.4223	0.0000	1.3550	0.0000	1.2002

1	5	4	70.0000	35.0000	3.4223	0.0000	1.3550	0.0000	1.2002
3	5	4	70.0000	35.0000	3.4223	0.0000	1.3550	0.0000	1.2002
5	1	7	70.0000	35.0000	3.4223	0.0000	1.3550	0.0000	1.2002
1	3	5	57.3353	41.0012	1.0609	0.0000	1.3000	0.0000	3.0000
3	3	5	83.9753	31.0715	3.5590	0.0000	0.8161	0.0000	1.1776
2	3	5	89.8843	17.5000	3.3660	0.0000	2.0000	0.0000	2.0734
2	6	2	0.0000	49.8261	0.2093	0.0000	2.0870	0.0000	2.2895
2	2	6	0.0000	39.7818	3.1505	0.0000	1.1296	0.0000	1.1110
6	2	6	0.0000	0.5047	0.8000	0.0000	0.8933	0.0000	4.6650
2	6	6	0.0000	8.7037	0.0827	0.0000	3.5597	0.0000	1.1198
3	6	3	0.0000	9.2317	0.1000	0.0000	1.0000	0.0000	1.0920
6	3	6	0.0008	25.0000	8.0000	0.0000	1.0000	0.0000	3.0000
2	3	6	66.0423	5.0000	1.0000	0.0000	1.0000	0.0000	1.2500
2	6	3	0.0000	0.5000	0.1000	0.0000	1.0000	0.0000	3.0000
3	3	6	70.0000	20.0000	1.0000	0.0000	1.0000	0.0000	1.2500
3	7	3	90.0000	18.4167	0.6799	-8.0000	0.1310	0.0000	2.2321
2	3	7	72.6004	9.6150	0.8905	0.0000	3.5473	0.0000	1.0400
3	3	7	60.0000	40.0000	4.0000	0.0000	1.0000	0.0000	1.0400
3	2	7	0.0000	10.0000	1.0000	0.0000	1.0000	0.0000	1.0400
6	3	7	41.0995	3.2207	7.3523	0.0000	0.1101	0.0000	1.0947
7	3	7	62.1312	7.5931	0.1000	0.0000	0.5154	0.0000	2.1744
1	3	7	74.1394	8.5687	1.7132	0.0000	-0.6553	0.0000	2.2323
2	7	3	75.0000	25.0000	2.0000	0.0000	1.0000	0.0000	1.2500
3	7	7	70.0000	25.0000	2.0000	0.0000	1.0000	0.0000	1.2500
1	1	9	68.2454	30.7089	7.3636	0.0000	0.9453	0.0000	3.0000
9	1	9	77.8443	49.0744	5.9913	0.0000	0.7835	0.0000	2.3020
1	9	1	0.0000	19.9962	3.2299	0.0000	2.1012	0.0000	1.1537
1	9	9	0.0000	25.0000	1.0000	0.0000	1.0000	0.0000	1.0400
2	1	9	57.2103	16.7391	2.8117	0.0000	1.0000	0.0000	1.0541
3	1	9	70.0000	25.0000	2.0000	0.0000	1.0000	0.0000	1.2500
1	4	9	74.3007	37.3795	6.7886	0.0000	1.4103	0.0000	3.0000
4	1	9	80.0406	45.0000	7.5000	0.0000	1.4103	0.0000	1.2468
9	7	9	100.0000	28.6831	3.7108	0.0000	2.4781	0.0000	2.3177
9	10	9	95.8799	25.0190	3.8255	0.0000	2.7415	0.0000	2.7526
2	11	2	25.9881	0.0100	1.8827	0.0000	0.6581	0.0000	1.1500
11	2	11	0.0000	3.6249	1.0000	0.0000	1.0000	0.0000	1.2500
3	11	3	12.9548	0.0000	2.9637	0.0000	2.1366	0.0000	1.0377
3	3	11	94.5107	15.5414	1.4415	0.0000	0.8277	0.0000	1.8911
11	3	11	38.8627	0.1417	0.2500	0.0000	2.4952	0.0000	1.0543
1	3	11	96.0884	23.6115	5.0000	0.0000	3.8782	0.0000	1.1267
2	12	2	25.9881	0.0100	1.8827	0.0000	0.6581	0.0000	1.1500
12	2	12	0.0000	3.6249	1.0000	0.0000	1.0000	0.0000	1.2500
3	1	12	97.3024	16.0013	5.0000	0.0000	0.0100	0.0000	4.0000
3	12	3	5.0901	0.0000	2.2094	0.0000	2.6087	0.0000	1.0736
3	3	12	83.5164	12.0682	0.5000	0.0000	0.1350	0.0000	3.2890
1	3	12	100.0000	24.9345	4.1297	0.0000	4.0000	0.0000	1.2192
3	1	11	88.4731	0.4260	0.7999	0.0000	0.9856	0.0000	1.9800
12	3	12	120.8627	50.1417	0.2500	0.0000	2.4952	0.0000	1.0543

```
67    ! Nr of torsions;at1;at2;at3;at4;;V1;V2;V3;V2(BO);vconj;n.u;n
 1  1  1  1  -0.2500  11.5822   0.1879  -4.7057  -2.2047   0.0000   0.0000
 1  1  1  2  -0.2500  31.2596   0.1709  -4.6391  -1.9002   0.0000   0.0000
 2  1  1  2  -0.1770  30.0252   0.4340  -5.0019  -2.0697   0.0000   0.0000
 1  1  1  3  -0.7098  22.2951   0.0060  -2.5000  -2.1688   0.0000   0.0000
 2  1  1  3  -0.3568  22.6472   0.6045  -4.0088  -1.0000   0.0000   0.0000
 3  1  1  3  -0.0528   6.8150   0.7498  -5.0913  -1.0000   0.0000   0.0000
 1  1  3  1   2.0007  25.5641  -0.0608  -2.6456  -1.1766   0.0000   0.0000
 1  1  3  2  -1.1953  42.1545  -1.0000  -8.0821  -1.0000   0.0000   0.0000
 2  1  3  1  -0.9284  34.3952   0.7285  -2.5440  -2.4641   0.0000   0.0000
 2  1  3  2  -2.5000  79.6980   1.0000  -3.5697  -2.7501   0.0000   0.0000
 1  1  3  3  -0.0179   5.0603  -0.1894  -2.5000  -2.0399   0.0000   0.0000
 2  1  3  3  -0.5583  80.0000   1.0000  -4.4000  -3.0000   0.0000   0.0000
 3  1  3  1  -2.5000  76.0427  -0.0141  -3.7586  -2.9000   0.0000   0.0000
 3  1  3  2   0.0345  78.9586  -0.6810  -4.1777  -3.0000   0.0000   0.0000
 3  1  3  3  -2.5000  66.3525   0.3986  -3.0293  -3.0000   0.0000   0.0000
 1  3  3  1   2.5000  -0.5332   1.0000  -3.5096  -2.9000   0.0000   0.0000
 1  3  3  2  -2.5000   3.3219   0.7180  -5.2021  -2.9330   0.0000   0.0000
 2  3  3  2   2.2500  -6.2288   1.0000  -2.6189  -1.0000   0.0000   0.0000
 1  3  3  3   0.0531 -17.3983   1.0000  -2.5000  -2.1584   0.0000   0.0000
 2  3  3  3   0.4723 -12.4144  -1.0000  -2.5000  -1.0000   0.0000   0.0000
 3  3  3  3  -2.5000 -25.0000   1.0000  -2.5000  -1.0000   0.0000   0.0000
 0  1  2  0   0.0000   0.0000   0.0000   0.0000   0.0000   0.0000   0.0000
 0  2  2  0   0.0000   0.0000   0.0000   0.0000   0.0000   0.0000   0.0000
 0  2  3  0   0.0000   0.1000   0.0200  -2.5415   0.0000   0.0000   0.0000
 0  1  1  0   0.0000  50.0000   0.3000  -4.0000  -2.0000   0.0000   0.0000
 0  3  3  0   0.5511  25.4150   1.1330  -5.1903  -1.0000   0.0000   0.0000
 0  1  4  0   1.7932 141.5515   0.9686  -4.2368  -1.9727   0.0000   0.0000
 0  2  4  0  -1.5000   0.1032   0.0100  -5.0965   0.0000   0.0000   0.0000
 0  3  4  0   1.1397  61.3225   0.5139  -3.8507  -2.7831   0.0000   0.0000
 0  4  4  0   0.7265  44.3155   1.0000  -4.4046  -2.0000   0.0000   0.0000
 4  1  4  4  -0.0949   8.7582   0.3310  -7.9430  -2.0000   0.0000   0.0000
 0  1  5  0   4.0885  78.7058   0.1174  -2.1639   0.0000   0.0000   0.0000
 0  5  5  0  -0.0170 -56.0786   0.6132  -2.2092   0.0000   0.0000   0.0000
 0  2  5  0   0.0000   0.0000   0.0000   0.0000   0.0000   0.0000   0.0000
 0  6  6  0   0.0000   0.0000   0.1200  -2.4426   0.0000   0.0000   0.0000
 0  2  6  0   0.0000   0.0000   0.1200  -2.4847   0.0000   0.0000   0.0000
 0  3  6  0   0.0000   0.0000   0.1200  -2.4703   0.0000   0.0000   0.0000
 1  1  1  7   0.0000  19.3871   0.0103 -25.5765  -1.7255   0.0000   0.0000
 7  1  1  7   0.0000  80.5586   0.1104  -8.0928  -1.7255   0.0000   0.0000
 0  1  7  0   4.0000  45.8264   0.9000  -4.0000   0.0000   0.0000   0.0000
 0  7  7  0   4.0000  45.8264   0.9000  -4.0000   0.0000   0.0000   0.0000
 2  1  3  7  -1.5000  18.9285   0.3649  -6.1208   0.0000   0.0000   0.0000
 2  3  7  3   1.5000  -1.0000   0.2575  -6.2100   0.0000   0.0000   0.0000
 1  3  7  3  -1.4375  -0.8700   0.9861  -2.5424   0.0000   0.0000   0.0000
 7  3  7  3  -1.5000  21.5086  -1.0000  -4.8869   0.0000   0.0000   0.0000
 1  1  1  9   0.5000   0.1000   0.4683 -11.5274  -1.7255   0.0000   0.0000
 2  1  1  9   0.0000  49.3871   0.2000 -10.5765  -1.7255   0.0000   0.0000
```

```
9  1  1  9  -0.5000  95.4727  -0.2080  -4.8579  -1.7255   0.0000   0.0000
0  1  9  0   1.0000  50.0000   0.9000  -3.0000   0.0000   0.0000   0.0000
0  9  9  0   1.0000  50.0000   0.9000  -3.0000   0.0000   0.0000   0.0000
1  1  3 11   0.0000  50.0000   0.2000  -4.0000   0.0000   0.0000   0.0000
2  1  3 11   0.0000  50.0000   0.2000  -4.0000   0.0000   0.0000   0.0000
3  1  3 11   0.0000  50.0000   0.2000  -4.0000   0.0000   0.0000   0.0000
1  1  3 12   0.0000  50.0000   0.2000  -4.0000   0.0000   0.0000   0.0000
2  1  3 12   0.0000  50.0000   0.2000  -4.0000   0.0000   0.0000   0.0000
3  1  3 12   0.0000  50.0000   0.2000  -4.0000   0.0000   0.0000   0.0000
0  1  5  0   4.0885  78.7058   0.1174  -2.1639   0.0000   0.0000   0.0000
0  2  5  0   0.0000   0.0000   0.0000   0.0000   0.0000   0.0000   0.0000
5  5  5  5   2.4661  71.9719   0.0100  -8.0000   0.0000   0.0000   0.0000
2  3  5  3   2.5000   2.5000   0.2237 -10.0000   0.0000   0.0000   0.0000
0  3  5  0   0.5000  50.0000   0.5000  -8.0000   0.0000   0.0000   0.0000
3  5  5  5   0.2500  90.0000   0.5000  -6.0000   0.0000   0.0000   0.0000
3  5  5  3   0.2500  90.0000   0.5000  -6.0000   0.0000   0.0000   0.0000
1  5  5  1   0.0000  50.0000   0.0000  -8.0000   0.0000   0.0000   0.0000
1  5  5  2   0.0000  50.0000   0.0000  -8.0000   0.0000   0.0000   0.0000
2  5  5  2   0.0000  50.0000   0.0000  -8.0000   0.0000   0.0000   0.0000
1  1  1  6   0.0000   5.0000   0.4000  -6.0000   0.0000   0.0000   0.0000
5    ! Nr of hydrogen bonds
3  2  3   1.9682  -4.4628   1.7976   3.0000
3  2  5   2.5000  -1.0000   1.4500  19.5000
5  2  3   2.5000  -1.0000   1.4500  19.5000
5  2  5   2.5000  -2.0000   1.4500  19.5000
1  2  3   1.7547  -0.2589   1.4500  19.5000
```
